# The Simplified Diet for PKU: Practices of Swedish Metabolic Dietitians

**DOI:** 10.3390/nu18111835

**Published:** 2026-06-05

**Authors:** Marika Kanthe, Camilla Widenberg Törnquist, Tom J. de Koning

**Affiliations:** 1 Section for Pediatrics, Department of Clinical Sciences, Lund University, 221 00 Lund, Sweden; tom.j_de_koning@med.lu.se; 2 Department of Pediatrics, Skane University Hospital, 221 85 Lund, Sweden; 3 Theme Women’s Health and Allied Health Professionals, Medical Unit for Allied Health Professionals, Section Pediatric Clinical Nutrition, Karolinska University Hospital, 171 76 Solna, Sweden; camilla.widenberg-tornquist@regionstockholm.se

**Keywords:** phenylketonuria (PKU), dietary management, simplified diet

## Abstract

**Background**: Dietary management of phenylketonuria (PKU) focuses on restricting phenylalanine (Phe) intake. The European PKU guidelines and the PKU Dietary Handbook recommend a simplified PKU diet, allowing unrestricted consumption of many low-Phe foods, called *free* foods. While this approach may reduce the treatment burden for patients, its implementation varies. This study investigated practices of Swedish metabolic dietitians regarding the simplified diet and the use of free foods for classical PKU (cPKU) and compared these with European recommendations. **Methods**: A survey was distributed to all metabolic dietitians in Sweden. The survey included questions on professional experience, the use of free foods and the classification of 135 low-protein food items as never, sometimes or always counted in cPKU. Data were summarised descriptively. **Results**: All 13 eligible dietitians participated. The use of free foods was recommended by 8/13 dietitians. Of foods classified as free in the PKU Handbook, about one third were commonly restricted in Swedish PKU practice. For 39% of the foods surveyed, no single response option (never, sometimes or always counted) reached >50%, indicating variation in practice. Classification of individual foods partially aligned with their Phe content, but portion size and concerns about excessive Phe intake also influenced advice. **Conclusions**: Significant variation exists in the dietary management of PKU in Sweden, and the simplified diet approach is not consistently implemented. Dietitians’ concerns about the safety of increased Phe intake from free foods play a central role in this. These findings highlight challenges in incorporating international guidelines into national practice and underscore the need for further research to address dietitians’ safety concerns related to the simplified diet.

## 1. Introduction

Phenylketonuria (PKU, OMIM #261600) is an autosomal recessive disorder caused by deficiency of the enzyme phenylalanine hydroxylase (PAH, EC 1.14.16.1). The role of PAH is to convert phenylalanine (Phe) into tyrosine, and deficiency of the enzyme results in an accumulation of Phe in body fluids, which can result in extensive neurological damage [1].

The mainstay of PKU treatment is a Phe-restricted diet. Early initiation of dietary treatment effectively prevents the symptoms of classical PKU (cPKU), such as intellectual disability, behavioural problems and epilepsy [2]. Pharmacological treatments are also available for selected patients.

### 1.1. PKU in Sweden

The incidence of PKU in Sweden is approximately 1 in 14,000 live births, similar to other European countries [3,4]. Based on genotype data, 42% of Swedish patients have cPKU compared with 62% of patients globally [4]. Since 2024, patients with inherited metabolic disorders, including PKU, are cared for at three centres for national specialised medical care: Sahlgrenska University Hospital in Gothenburg, Skane University Hospital in Lund and Karolinska University Hospital in Stockholm. At the time of this study, 13 registered dietitians were working in inherited metabolic disorders across these three specialist centres.

The Swedish Registry for Inherited Metabolic Diseases includes 379 patients with PKU in its quality component (March 2026). Data from the registry shows that in 2025, 86% of all Swedish PKU patients aged 0–12 years had mean blood Phe levels within the target range (120–360 µmol/L). Among patients aged 13–17 years and ≥18 years, 87% and 59%, respectively, had blood Phe levels within the target range of 120–600 µmol/L [5].

### 1.2. Dietary Management of PKU

The key principle of the PKU diet is a strict restriction of protein from food, to limit the intake of Phe [6]. The PKU diet therefore consists mainly of foods with a low-protein content such as vegetables, fruit, fats, starch, and low-protein special foods such as pasta, rice, flour and milk replacement. Meat, fish, eggs, milk and dairy products, nuts, grains and pulses cannot be consumed due to their high protein content. An individual’s daily Phe tolerance is largely genotype dependent and remains relatively constant throughout life. Individual tolerance is determined by titrating protein/Phe intake to blood Phe levels, which informs the daily allowance prescribed by the metabolic dietitian [6]. Parents, and later patients, are instructed to weigh and measure the foods that contribute to their daily allowance (mainly potatoes, vegetables, and small amounts of cereal and dairy products) and keep track of the intake on a day-to-day basis. The limited selection of suitable low-protein foods and the need for constant planning and monitoring of intake leads to diminished flexibility in daily life, and often to social stigma [7]. It is well-known that the adherence to dietary treatment frequently deteriorates in adolescence and may remain low in adulthood [8], resulting in persistently elevated blood Phe levels. This is also evident in data from the Swedish registry, where fewer adult patients have mean blood Phe levels within the target range compared with younger patients [5].

### 1.3. Free Foods

*Free* foods are defined as those that are unlikely to affect blood Phe levels in PKU and may therefore be consumed freely, without calculating and monitoring the intake. However, views on which foods actually qualify as free vary. This was evident from a 2009 survey on PKU dietary treatment in ten centres across Europe, with some centres reporting cut-offs for free foods of between 10 and 25 mg Phe/100 g, whereas other centres allowed only foods inherently very low in Phe, such as oils, sugar, honey, spices and vinegar. Fruits and vegetables constituted a separate category in the survey, and practices ranged from considering all free to regarding none as free, with some centres at the time using cut-offs of between 5 mg per serving and 50 mg/100 g [9]. Similarly, a more recent Italian multicentre survey reported practices ranging from allowing all fruits and vegetables without restriction, to requiring patients to count all foods [10].

### 1.4. Free Foods in the Simplified PKU Diet

In 2020, The PKU Dietary Handbook was published as a companion to the European PKU guidelines [6]. It states that fruits and vegetables (except potatoes) with a maximum Phe content of 75 mg/100 g can be consumed without restriction by all PKU patients, regardless of what system for protein or Phe calculation is used. In that same year, UK consensus statements on the allocation of foods in the low-Phe diet were published by the British Inherited Metabolic Disease Group Dietitians Group (BIMDG-DG) [11]. These statements cover all relevant food groups and state that in addition to fruits and vegetables, many manufactured special foods and other naturally low-Phe foods may be consumed freely as well. This approach, often called the simplified PKU diet, reduces the number of foods requiring calculation and may therefore lessen the treatment burden for patients and families. Earlier studies conducted in the UK, Australia, Switzerland and Germany suggested that adopting such a simplified diet approach does not compromise metabolic control compared to traditional PKU dietary management, and the concept has been in clinical use for many years [12,13,14,15,16].

### 1.5. Prior Knowledge of Practices in Sweden

From meetings within the Section for Dietitians in Inherited Metabolic Disorders of the Swedish Association of Registered Dietitians, we know that the definition and use of free foods for PKU patients vary within the dietitians’ group, ranging from advising patients to count protein or Phe from all foods to allowing a variety of low-Phe foods without restriction. However, detailed insight into the views on which specific foods are considered free by Swedish PKU dietitians is lacking at the moment. Local lists of free foods exist, but the extent of their usage in daily practice is unclear. Protein or Phe intake is monitored directly; that is, an exchange system with pre-calculated food portions providing a fixed amount of Phe is not used.

### 1.6. Aim

Considering the apparent gap between the PKU Dietary Handbook and the UK consensus statements on the one hand, and the daily clinical practices of metabolic dietitians in Sweden and other European countries on the other, the aim of this study was to explore the views and practices of Swedish metabolic dietitians regarding free foods in PKU, and compare them with the current European recommendations. This study examines national practices on a food-item level, and we are certain the results of our study provide valuable input for further discussions on harmonisation of the dietary treatment in PKU.

## 2. Materials and Methods

An online survey was distributed to all registered dietitians in Sweden who manage patients with PKU in both paediatric and adult clinics. The survey was administered via REDCap, a secure, web-based platform hosted locally by Lund University. Participants completed the survey anonymously, with respect to which PKU centre each respondent belonged to.

The survey focused on dietary advice given to patients aged six years or older with cPKU. We looked at cPKU specifically, since these patients have the lowest Phe tolerance and any differences in practice would be most obvious for this group. For the purposes of the survey, cPKU was defined as PAH deficiency with either (i) two null PAH mutations or (ii) a documented cPKU severity classification in medical records. Advice for children under six was excluded, as prior discussions indicated that Swedish dietitians often adopt a stricter approach in early childhood than at later ages.

The first part of the survey contained six questions addressing the respondents’ work experience and practices, including whether they advise their patients to count protein or Phe, and their use of free foods. The second part focused on the dietitians’ advice on 135 individual food items suitable for a Swedish low-protein diet. The food items comprised fresh fruits and berries (n = 34), dried fruits (n = 8), vegetables (root and cruciferous vegetables, mushrooms, leafy greens, potatoes and corn, n = 60), peas and edible pods (n = 7), manufactured low-protein products (e.g., milk replacement, pasta, rice, flour and crispbread, n = 12), and other naturally low-protein foods (n = 14). According to the PKU Dietary Handbook, 99 of these 135 food items were foods that would be considered free, four were foods that would be considered free if the protein content was below a certain cut-off, and 32 were foods that would be calculated as part of the daily allowance. Foods containing negligible amounts of Phe (e.g., sugar, honey, oils, vinegar) were not included in the survey.

For each of the 135 food items, respondents answered the question:

“Do you advise your patients aged six years and over with classical PKU to count this food item towards the daily protein allowance?”

Response options were: “Never”, “Sometimes”, “Always”, “I would discourage against this food item”, and “I have never given advice on this food item”.

A comment field at the end of the survey invited respondents to share their thoughts on their clinical practice or the survey content.

### 2.1. Data Analysis

Data were analysed with descriptive statistics. For the second part of the survey, the primary outcome was the most frequently selected response option for each of the 135 food items. Response options “Never”, “Sometimes” and “Always” were treated as ordinal categories, and when ties occurred between “Sometimes” and either adjacent response category (“Never” or “Always”), the food item was classified into the “Sometimes” category. Food items for which a majority of participants selected the response “I have never given advice on this food item” were excluded from further descriptive analysis. For items where this response was common but did not exceed 50%, the most frequent of the other response options was reported.

Phe content was calculated for all food items by collating and averaging data from multiple general and PKU-specific sources before comparison with survey responses [17,18,19,20,21,22,23].

### 2.2. Ethical Considerations

Since the survey was anonymous and no personal or identifying data were collected, according to Swedish regulations, formal ethical approval was not required.

## 3. Results

All 13 registered dietitians working in inherited metabolic disorders in Sweden at the time participated in the survey. Surveys were completed between November 2024 and January 2025. General questions in part 1 were fully completed by all dietitians. Ninety-four per cent (127/135) of food-related questions in part 2 were answered by all respondents; the remaining eight questions had one or two missing responses.

### 3.1. Part 1: General Questions

Of the 13 dietitians, eight worked exclusively in paediatrics (0–17 years), three in adult care, and two treated both children and adults. Over half (7/13) of the dietitians had more than ten years’ experience in managing PKU patients, three had five to ten years’ experience, and three had less than five years.

Regarding methods for protein counting, eight dietitians reported that their cPKU patients aged six years and older were advised to count protein. The remaining five indicated mixed practice, though most of their patients also counted protein, as opposed to Phe. None reported that all or most of their patients were advised to count Phe. Several dietitians emphasised that the choice of recommending protein or Phe calculation depended either on the patient’s capability (n = 4) or their blood Phe control (n = 3), implying that Phe calculation is more demanding but also more accurate.

Free foods were used routinely by eight respondents, not used at all by two, and used with selected patients only by three. As already mentioned, free foods excluded protein-free items such as sugar, honey, oils and vinegar. The dietitians who said they used free foods were also asked whether they had expanded the range of free foods in their advice to patients during the past five years. To this, one respondent answered yes, four no, and six were unsure.

### 3.2. Part 2: Classification of Low-Protein Food Items

In part 2 of the survey, respondents answered the question “Do you advise your patients aged six years and over with classical PKU to count this food item towards the daily protein allowance?”.

#### 3.2.1. Items Unfamiliar in Clinical Practice

For 11 of the 135 food items (8%), the majority of respondents selected the response option *I have never given advice on this food item.* The items included açai berries, cavolo nero, goji berries, vine leaves, nettles, pak choi, prickly pears, rosehip, runner beans, sauerkraut, and seaweed as used in sushi. As the majority of dietitians had no experience of giving advice on these foods, they were excluded from further analyses based on most common responses.

#### 3.2.2. Classification of Foods Considered Free in the Simplified Diet

Of the 96 food items in our survey that could be allocated without restriction according to the BIMDG-DG consensus statements (items unfamiliar in practice excluded), one-third (34 items, 35%) had the most common response of *Sometimes* or *Always* in the survey. Examples included beetroot, olives, cabbage (*Sometimes*), mushrooms, avocado and bananas (*Always*) (Table 1). On the other hand, 8/35 (23%) of the food items in the survey that were classified as “allocated as part of the protein exchange system” in the BIMDG-DG statements [11] had the most common response of *Never*, and thus were often regarded as free; these were mustard, rocket, stock, soy sauce, passion fruit, fresh figs, vegan mayonnaise and hamburger dressing (the latter two requiring counting if protein content is >1 g/100 g).

#### 3.2.3. Distribution of Responses

About 90% of all fresh fruits and berries and 75% of low-protein special foods and naturally low-protein foods in the survey were categorised as *Never* counted. One hundred percent of peas and edible pods fell into the *Always* group. As for the vegetables, about one third was allocated to each of the response groups *Never*, *Sometimes* and *Always*, making vegetables the main food category in the *Sometimes* group. Three out of five dried fruit items also fell into the *Sometimes* group, see Table S1.

For 49 of 124 food items (39%), no response option achieved a majority, reflecting a lack of consistency of advice among the dietitians. Marked variation in responses was observed for many individual items frequently used in the PKU diet; aubergine, chard, white cabbage, leeks, soy sauce, orange juice, Loprofin Drink LQ, courgette, and tomato purée each received at least three *Never* and three *Always* responses, illustrating considerable variation in their use in clinical practice (Table 2). All in all, 53% (71/135) of all surveyed foods had a response range from *Never* to *Always*. In contrast, a small number of food items, namely cucumber, margarine, ginger, butter and coconut fat showed a high level of agreement; 11 or 12 respondents selected *Never* for each.

#### 3.2.4. Association Between Responses and Protein/Phe Content

**Figure 1:**
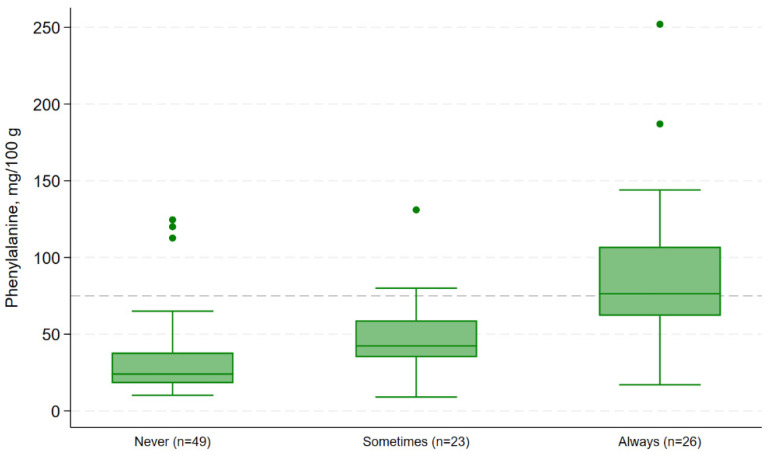
Phenylalanine content of fruits and vegetables grouped by most common answer. The horizontal dashed line indicates the Phe cut-off (75 mg/100 g) for free fruits and vegetables.

To examine how the reported classification of the surveyed foods aligned with their protein and Phe content, fruits and vegetables were grouped by their most common response, and these groups were then compared in terms of protein and Phe content. Fruits and vegetables in the *Never* group had a mean Phe content of 33 mg/100 g (n = 49; range 10–125 mg/100 g) and a mean protein content of 1.0 g/100 g (0.4–3.1 g/100 g). Foods in the *Sometimes* group contained a mean of 48 mg Phe/100 g (n = 23; range 9–131 mg/100 g) and 1.7 g protein/100 g (0.7–3.9 g/100 g). The *Always* group had a mean Phe content of 89 mg/100 g (n = 26; range 17–252 mg/100 g) and a mean protein content of 2.6 g/100 g (0.5–6.8 g/100 g). This means that the Phe and protein content generally tracked with the advice to count an item or not, but that substantial overlap existed between response category groups (Figure 1). Eighty-seven percent of foods in the *Sometimes* group (20/23) and 46% in the *Always* group (12/26) had a Phe content of <76 mg/100 g.

#### 3.2.5. Items Discouraged for PKU

Ten items (7%) were marked as discouraged for cPKU patients by at least one respondent. Broad beans were the most frequently discouraged (n = 3). Single respondents discouraged inclusion of green beans, peas, wax beans (n = 2), seaweed, runner beans, mangetout, sugar snap peas, raisins, and dried cranberries (n = 1) into the diet for cPKU. Of these, green beans, wax beans, raisins and dried cranberries are classified as free in the PKU Dietary Handbook [6].

### 3.3. Information from the Comments Field

Free text comments were provided by ten respondents and offered additional insight into how advice about counting specific foods is given in practice. Five out of ten dietitians noted that recommendations usually depend on the amount a patient is likely to consume; foods considered free in small portions may need to be counted when eaten in larger quantities, or by patients with poor blood Phe control. Relating to the use of free foods, one respondent reported reliance on established local “free food lists” and another typically informed families about which foods do not need to be counted, assuming that the remaining items will be counted if used in the diet. Respondents further emphasised the need to adapt advice on protein counting to individual circumstances, including eating habits, parental capacity, treatment goals, severe phenotypes, or during pregnancy.

## 4. Discussion

The aim of this study was to examine the practices of Swedish metabolic dietitians regarding the use of free foods in PKU and compare these practices to current European recommendations. We think our work offers an interesting perspective on the real-world national implementation of international dietary guidelines, and insight into how specific food items in the PKU diet are classified by a group of highly experienced metabolic dietitians. We identified substantial differences in the clinical practice of Swedish PKU dietitians compared to the European guidelines, as well as within the group itself.

Interestingly, while fruits and berries were generally considered free, and peas and edible pods were consistently counted, over half of the surveyed food items had responses spanning from *Never* to *Always* counted. Along with the fact that for more than one third of the items, no single response option achieved a majority, this shows that despite the basic principles of the PKU diet being the same universally, practical application may vary considerably. Different kinds of discrepancies in the practical application of low-protein diets are not unique for PKU. Several European survey studies on other inherited metabolic disorders have shown heterogenous dietary practices [24,25,26], and a 2024 Italian survey on dietary practices for PKU reported marked differences in the use of free foods across national centres [10]. Notably, recent multicentre European data and registry data from Sweden show comparable metabolic control across countries, suggesting that variation in clinical practice does not necessarily translate into differences in biochemical outcomes for PKU patients [5,8].

Early studies from the UK, later supported by research from several other countries, showed that allowing unrestricted intake of fruits and vegetables containing up to 75 mg Phe/100 g did not impair metabolic control in either moderate or severe PKU [12,13,14,15,16,27,28]. These findings underpin the simplified diet recommendations in the European PKU guidelines and the PKU Dietary Handbook [2,6]. Nevertheless, implementation of the simplified concept remains incomplete, as our data shows. In our survey, five out of thirteen respondents stated that they either did not use free foods at all or used it only selectively or in small portions. Again, our results align with the 2024 Italian survey on PKU dietary practices, where 5/13 treatment centres advised patients to count all foods [10]. Even though more than half of the Swedish metabolic dietitians used free foods in their clinical practice for PKU, only one dietitian indicated that the range of free foods used for their PKU patients had expanded since the PKU Dietary Handbook was published.

Apart from the observation that a large proportion of foods categorised as *Sometimes* or *Always* counted contained less than 76 mg Phe per 100 g, comparison of Phe and protein content of surveyed fruits and vegetables revealed that many foods in the *Sometimes* group had similar Phe and protein content to those in the *Never* group. Comments provided by respondents expressed the view that the added intake of Phe from free foods would impact blood Phe levels. This likely represents the “missing factor” underlying food classification, whereby foods expected to be consumed in larger quantities more frequently fell into the counted categories despite a low protein or Phe content.

Similar concerns about excessive Phe intake have been addressed in studies on the simplified diet, with different adaptations to resolve the issue; either reducing the participants’ Phe allowances when switching to the simplified diet, or adding a set lump sum for Phe from free foods to the daily calculation [14,29]. The European PKU guidelines and the PKU Dietary Handbook however do not advise reducing the allowance with the simplified diet. Instead, free foods are considered unlikely to affect metabolic control regardless of the amount consumed [2,6]. Our study suggests that the safety concerns among Swedish PKU dietitians may not be fully resolved by the existing evidence. The certainty of evidence supporting unrestricted consumption of low Phe fruits and vegetables was rated as low to very low in the systematic review underpinning the 2017 European guidelines [30]. Given the inherent challenges of conducting nutritional research in a rare disease context, this level of evidence is not unexpected and does not mean that the results should be dismissed. The simplified diet has been used successfully for many years in the UK [12,27]. Still, for dietitians with long-standing experience of strict Phe counting, transitioning to a simplified approach might be daunting. Beyond safety concerns, additional barriers may impede wider adoption of the simplified diet. Factors such as insufficient Phe analysis data, resistance from other team members, and ambiguous labelling on an extensive and constantly changing range of manufactured foods have been cited [11,29].

Acknowledging and addressing these concerns is necessary to facilitate broader and more uniform implementation of the simplified diet approach for PKU. We do believe that the Swedish metabolic dietitians’ group is well placed to address these issues moving forward. Furthermore, the detailed knowledge of dietary practice at the food-item level gained from our study provides a unique opportunity to investigate how harmonising treatment advice and switching to a simplified diet would affect the metabolic control and quality of life of Swedish PKU patients.

## 5. Conclusions

This study included all dietitians working clinically with PKU patients in Sweden, representing a national cohort with substantial experience in the dietary management of PKU. Our findings show that the concept of unrestricted intake of low-Phe free foods as recommended in the PKU Dietary Handbook has not been fully implemented in Sweden. The reasons for this are likely multifactorial but concerns about the safety of increased phenylalanine intake from unrestricted free foods play a central role. National implementation of the simplified diet could eliminate any ineffective restrictions currently in use, improve the clarity of dietary advice, and hopefully lead to a better quality of life for Swedish PKU patients. It also has the added benefit of supporting healthy dietary choices.

## Figures and Tables

**Table 1:** Foods classified as free by the BIMDG-DG consensus statements that had a most common response of “Sometimes” or “Always” counted in the survey of Swedish metabolic dietitians.

Sometimes	Always
Apricots, dried	Avocado
Bananas	Baby corn
Beetroot	Chanterelle mushrooms
Chard	Cranberries
Globe artichoke	Dates
Jerusalem artichoke	Fennel
Okra	Green beans
Olives, black	Jackfruit
Olives, green	Mushrooms, fresh
Orange juice	Mushrooms, tinned
Prunes	Tomato purée
Pumpkin	Tomato sauce
Raisins	Wax beans
Swede	
Sweet potato	
White cabbage	

**Table 2:** Food items with the most diverse responses.

Food Item	Never Counted	Sometimes Counted	Always Counted
Leeks	7	2	3
Soy sauce	6	0	5
Courgette	5	4	3
Tomato purée	4	3	5
Aubergine	4	3	3
Orange juice	4	5	3
Loprofin PKU Drink LQ	4	6	3
Chard	3	5	3
White cabbage	3	4	3

## Data Availability

The raw data supporting the conclusions of this article will be made available by the authors on request.

## Supplementary Materials

The following supporting information can be downloaded at https://www.mdpi.com/article/10.3390/nu18111835/s1, Table S1: Results for surveyed food items.
