# Supplementary material for: The Simplified Diet for PKU: Practices of Swedish Metabolic Dietitians
Source: Nutrients. 2026 Jun 5;18(11):1835. doi: 10.3390/nu18111835 (PMC13258811; doi:10.3390/nu18111835)
Supplement: Supplementary file 1 [file nutrients-18-01835-s001.zip › Table S1.pdf]

Table S1. Results for surveyed food items. Averaged protein and phenylalanine content and classification according to the BIMDG-DG [11] included.

Food item	Free[a]	Phe content[b] mg/100 g	Protein content[b] g/100 g	Respondents (n)	No experience		Never		Sometimes		Always		Discouraged		Most common answer
					(n)	(%)	(n)	(%)	(n)	(%)	(n)	(%)	(n)	(%)	
Potatoes	No	66	1,6	13	0	0	0	0	3	23	10	**77**	0	0	Always
Corn	No	141	3,0	13	0	0	1	8	3	23	9	**69**	0	0	Always
Glutis pasta[c]	No	191	4,0	13	3	23	0	0	1	8	9	**69**	0	0	Always
Schär crispbread[d]	No	186	3,6	13	1	8	0	0	3	23	9	**69**	0	0	Always
Broad beans	No	252	6,8	13	1	8	0	0	1	8	8	**62**	3	23	Always
Green beans	Yes	65	1,8	13	1	8	0	0	2	15	8	**62**	2	15	Always
Mushrooms, fresh	Yes	89	2,8	13	0	0	1	8	4	31	8	**62**	0	0	Always
Mushrooms, tinned	Yes	76	1,9	13	1	8	1	8	3	23	8	**62**	0	0	Always
Peas	No	187	5,5	13	1	8	0	0	2	15	8	**62**	2	15	Always
Brussels sprouts	No	90	3,1	12	2	17	0	0	3	25	7	**58**	0	0	Always
Broccoli	No	86	3,2	11	0	0	0	0	5	46	6	**55**	0	0	Always
Avocado	Yes	61	1,8	13	3	23	0	0	3	23	7	**54**	0	0	Always
Baby corn	Yes	73	1,9	13	2	15	1	8	3	23	7	**54**	0	0	Always
Bean sprouts	No	107	3,0	13	3	23	0	0	3	23	7	**54**	0	0	Always
Cauliflower	No	87	2,7	13	1	8	1	8	4	31	7	**54**	0	0	Always
Spinach	No	144	2,5	13	2	15	1	8	3	23	7	**54**	0	0	Always
Sugar snap peas	No	74	3,3	13	2	15	0	0	3	23	7	**54**	1	8	Always
Wax beans	Yes	62	2,0	13	1	8	0	0	3	23	7	**54**	2	15	Always
Mangetout	No	77	3,5	12	2	17	0	0	3	25	6	**50**	1	8	Always
Chanterelle mushrooms	Yes	57	1,7	13	1	8	1	8	5	39	6	**46**	0	0	Always
Figs, dried	No	116	3,4	13	4	31	0	0	3	23	6	**46**	0	0	Always
Jackfruit	Yes	40	1,0	13	2	15	1	8	4	31	6	**46**	0	0	Always
Kale	No	134	2,9	13	2	15	0	0	5	39	6	**46**	0	0	Always
Tomato sauce	Yes	36	1,3	13	1	8	2	15	4	31	6	**46**	0	0	Always
Dates	Yes	63	2,1	13	4	31	0	0	4	31	5	**39**	0	0	Always

Food item	Free[a]	Phe content[b] mg/100 g	Protein content[b] g/100 g	Respondents (n)	No experience (n)	(%)	Never (n)	(%)	Sometimes (n)	(%)	Always (n)	(%)	Discouraged (n)	(%)	Most common answer
Gelatine	No	1800	86,5	13	3	23	2	15	3	23	5	**39**	0	0	Always
Tomato purée	Yes	86	3,7	13	1	8	4	31	3	23	5	**39**	0	0	Always
Cranberries	Yes	17	0,5	13	4	31	2	15	2	15	4	**31**	1	8	Always
Fennel	Yes	26	0,9	13	6	46	2	15	2	15	3	**23**	0	0	Always
Mayonnaise	No[e]	56	0,8	13	0	0	2	15	9	**69**	2	15	0	0	Sometimes
Asparagus	No	79	3,1	13	1	8	0	0	7	**54**	5	39	0	0	Sometimes
Beetroot	Yes	26	2,1	12	0	0	3	25	7	**58**	2	17	0	0	Sometimes
Celeriac	Yes	31	1,0	13	3	23	2	15	7	**54**	1	8	0	0	Sometimes
Parsnip	Yes	47	1,4	13	3	23	2	15	7	**54**	1	8	0	0	Sometimes
Sweet potato	Yes	42	1,2	13	1	8	0	0	7	**54**	5	39	0	0	Sometimes
Bananas	Yes	42	1,2	13	1	8	0	0	6	**46**	6	**46**	0	0	Always/Sometimes
Loprofin Drink LQ	No	10	0,4	13	0	0	4	31	6	**46**	3	23	0	0	Sometimes
Olives, black	Yes	39	0,9	13	2	15	1	8	6	**46**	4	31	0	0	Sometimes
Olives, green	Yes	39	0,9	13	2	15	2	15	6	**46**	3	23	0	0	Sometimes
Raisins	Yes	60	2,7	13	1	8	1	8	6	**46**	4	31	1	8	Sometimes
Red cabbage	Yes	44	1,3	13	4	31	2	15	6	**46**	1	8	0	0	Sometimes
Apricots, dried	Yes	50	3,9	13	3	23	0	0	5	**39**	5	**39**	0	0	Always/Sometimes
Bamboo shoots	No	51	1,2	13	3	23	1	8	5	**39**	4	31	0	0	Sometimes
Chard	Yes	131	1,9	13	2	15	3	23	5	**39**	3	23	0	0	Sometimes
Orange juice	Yes	9	0,7	13	1	8	4	31	5	**39**	3	23	0	0	Sometimes
Pumpkin	Yes	39	0,9	13	4	31	2	15	5	**39**	2	15	0	0	Sometimes
Swede	Yes	24	0,7	13	3	23	3	23	5	**39**	2	15	0	0	Sometimes
Prunes	Yes	52	2,3	13	5	39	0	0	4	**31**	4	**31**	0	0	Always/Sometimes
Aquafaba	No	55	1,4	13	6	46	0	0	4	**31**	3	23	0	0	Sometimes
Okra	Yes	80	3,6	13	6	46	0	0	4	**31**	3	23	0	0	Sometimes
White cabbage	Yes	35	1,1	13	3	23	3	23	4	**31**	3	23	0	0	Sometimes

Food item	Free[a]	Phe content[b] mg/100 g	Protein content[b] g/100 g	Respondents (n)	No experience		Never		Sometimes		Always		Discouraged		Most common answer
					(n)	(%)	(n)	(%)	(n)	(%)	(n)	(%)	(n)	(%)	
Daikon	Yes	31	1,8	13	5	39	4	**31**	4	**31**	0	0	0	0	Sometimes/Never
Globe artichoke	Yes	65	1,7	13	6	46	2	15	3	**23**	2	15	0	0	Sometimes
Jerusalem artichoke	Yes	59	1,6	13	6	46	2	15	3	**23**	2	15	0	0	Sometimes
Savoy cabbage	Yes	41	1,1	13	6	46	3	**23**	3	**23**	1	8	0	0	Sometimes/Never
Butter	Yes	30	0,6	13	1	8	12	**92**	0	0	0	0	0	0	Never
Cucumber	Yes	14	0,6	13	1	8	12	**92**	0	0	0	0	0	0	Never
Ginger	Yes	46	1,8	13	1	8	12	**92**	0	0	0	0	0	0	Never
Margarine	Yes	25	0,5	13	1	8	12	**92**	0	0	0	0	0	0	Never
Coconut fat	Yes	0	0,0	13	2	15	11	**85**	0	0	0	0	0	0	Never
Ketchup	Yes	52	2,0	13	0	0	11	**85**	2	15	0	0	0	0	Never
Blackberries	Yes	41	1,5	13	1	8	10	**77**	2	15	0	0	0	0	Never
Clementines	Yes	22	0,8	13	1	8	10	**77**	1	8	1	8	0	0	Never
Grapefruit	Yes	12	0,8	13	2	15	10	**77**	1	8	0	0	0	0	Never
Lettuce	Yes	45	1,0	13	1	8	10	**77**	1	8	1	8	0	0	Never
Loprofin Egg Replacement	Yes	5	0,3	13	1	8	10	**77**	1	8	1	8	0	0	Never
Mustard	No	240	5,7	13	2	15	10	**77**	1	8	0	0	0	0	Never
Pineapple	Yes	10	0,4	13	1	8	10	**77**	1	8	1	8	0	0	Never
Psyllium	Yes	0	1,2	13	0	0	10	**77**	3	23	0	0	0	0	Never
Taco spice mix	Yes	286	9,7	13	2	15	10	**77**	1	8	0	0	0	0	Never
Rhubarb	Yes	21	1,0	12	1	8	9	**75**	2	17	0	0	0	0	Never
Black- and redcurrants	Yes	44	1,5	13	3	23	9	**69**	1	8	0	0	0	0	Never
Blueberries	Yes	24	0,5	13	1	8	9	**69**	3	23	0	0	0	0	Never
Fate All-Purpose Mix[f]	Yes	4	0,3	13	0	0	9	**69**	3	23	1	8	0	0	Never
Kiwi fruit	Yes	38	1,0	13	1	8	9	**69**	2	15	1	8	0	0	Never
Lingonberries	Yes	15	0,5	13	2	15	9	**69**	2	15	0	0	0	0	Never
Loprofin Cereal	Yes	6	0,3	13	0	0	9	**69**	3	23	1	8	0	0	Never

Food item	Free[a]	Phe content[b] mg/100 g	Protein content[b] g/100 g	Respondents (n)	No experience		Never		Sometimes		Always		Discouraged		Most common answer
					(n)	(%)	(n)	(%)	(n)	(%)	(n)	(%)	(n)	(%)	
Loprofin Pasta	Yes	18	0,5	13	0	0	9	**69**	3	23	1	8	0	0	Never
Loprofin Rice	Yes	14	0,4	13	0	0	9	**69**	3	23	1	8	0	0	Never
Oranges	Yes	17	0,9	13	1	8	9	**69**	2	15	1	8	0	0	Never
Pofiber[g]	Yes	0	5,0	13	1	8	9	**69**	2	15	1	8	0	0	Never
Pomegranate	Yes	65	1,4	13	2	15	9	**69**	2	15	0	0	0	0	Never
Raspberries	Yes	36	1,2	13	1	8	9	**69**	3	23	0	0	0	0	Never
Rocket	No	113	3,1	13	1	8	9	**69**	1	8	2	15	0	0	Never
Tomato	Yes	24	0,9	13	1	8	9	**69**	2	15	1	8	0	0	Never
Stock	No	5	0,4	12	0	0	8	**67**	3	25	1	8	0	0	Never
Apples	Yes	11	0,4	13	1	8	8	**62**	3	23	1	8	0	0	Never
Cherries	Yes	24	1,4	13	3	23	8	**62**	2	15	0	0	0	0	Never
Lard	Yes	0	0,0	13	5	39	8	**62**	0	0	0	0	0	0	Never
Loprofin Crackers	Yes	10	0,4	13	0	0	8	**62**	4	31	1	8	0	0	Never
Mango	Yes	16	0,5	13	1	8	8	**62**	3	23	1	8	0	0	Never
Nectarines	Yes	19	1,1	13	1	8	8	**62**	3	23	1	8	0	0	Never
Peaches	Yes	19	0,9	13	1	8	8	**62**	4	31	0	0	0	0	Never
Pears	Yes	12	0,4	13	1	8	8	**62**	3	23	1	8	0	0	Never
Vegan mayonnaise	No[h]	13	0,3	13	1	8	8	**62**	3	23	1	8	0	0	Never
Glass noodles	Yes	10	0,2	12	0	0	7	**58**	4	33	1	8	0	0	Never
Cloudberries	Yes	39	1,3	13	4	31	7	**54**	2	15	0	0	0	0	Never
Gooseberries	Yes	24	0,8	13	5	39	7	**54**	1	8	0	0	0	0	Never
Grapes	Yes	12	0,6	13	1	8	7	**54**	3	23	2	15	0	0	Never
Laila's Flour Mix[i]	Yes	25	0,5	13	1	8	7	**54**	3	23	2	15	0	0	Never
Leeks	Yes	47	1,6	13	1	8	7	**54**	2	15	3	23	0	0	Never
Physalis	Yes	62	1,6	13	4	31	7	**54**	2	15	0	0	0	0	Never
Plums, fresh	Yes	13	0,7	13	1	8	7	**54**	5	39	0	0	0	0	Never

Food item	Free[a]	Phe content[b] mg/100 g	Protein content[b] g/100 g	Respondents (n)	No experience (n)	(%)	Never (n)	(%)	Sometimes (n)	(%)	Always (n)	(%)	Discouraged (n)	(%)	Most common answer
Radish	Yes	20	0,7	13	2	15	7	**54**	3	23	1	8	0	0	Never
Sharon fruit	Yes	23	0,8	13	3	23	7	**54**	3	23	0	0	0	0	Never
Strawberries	Yes	22	0,8	13	1	8	7	**54**	5	39	0	0	0	0	Never
Soy sauce	No[j]	281	6,5	12	1	8	6	**50**	0	0	5	42	0	0	Never
Apricots, fresh	Yes	31	1,0	13	4	31	6	**46**	2	15	1	8	0	0	Never
Capers	Yes	120	2,4	13	6	46	6	**46**	1	8	0	0	0	0	Never
Carrots	Yes	21	0,8	13	0	0	6	**46**	5	39	2	15	0	0	Never
Celery	Yes	13	0,6	13	3	23	6	**46**	3	23	1	8	0	0	Never
Melon	Yes	19	0,8	13	1	8	6	**46**	5	39	1	8	0	0	Never
Peppers	Yes	30	0,9	13	1	8	6	**46**	5	39	1	8	0	0	Never
Chinese cabbage	Yes	36	1,1	13	4	31	5	**39**	4	31	0	0	0	0	Never
Courgette	Yes	38	1,3	13	1	8	5	**39**	4	31	3	23	0	0	Never
Hamburger dressing[k]	No[e]	79	1,4	13	2	15	5	**39**	4	31	2	15	0	0	Never
Papaya	Yes	16	0,5	13	5	39	5	**39**	3	23	0	0	0	0	Never
Passion fruit	No	125	2,4	13	3	23	5	**39**	4	31	1	8	0	0	Never
Aubergine	Yes	37	1,0	13	3	23	4	**31**	3	23	3	23	0	0	Never
Chicory	Yes	25	0,6	13	6	46	4	**31**	2	15	1	8	0	0	Never
Kohlrabi	Yes	29	1,1	13	6	46	4	**31**	2	15	1	8	0	0	Never
Turnip	Yes	18	0,6	13	6	46	4	**31**	3	23	0	0	0	0	Never
Figs, fresh	No	32	0,7	13	6	46	3	**23**	2	15	2	15	0	0	Never
Water chestnuts	Yes	32	0,8	13	6	46	3	**23**	2	15	2	15	0	0	Never

a) Can be eaten without restriction according to [11]. b) Data collated and averaged from [17-23]. c) Based on corn flour, 4,0 g protein/100 g. d) Based on rice flour, 3,6 g protein/100 g. e) Counted unless protein <1 g/100 g. f) Yeast not included. g) Potato-based fibre product, 5,0 g protein/100 g. h) Counted if it contains exchange ingredients and >1,0 g protein/100 g. i) Gluten-free flour, <0,5 g of protein/100 g. j) Counted unless protein <1,5 g/100 g. k) Mayonnaise-based hamburger sauce, >1,0 g protein/100 g.

Phe: phenylalanine; No experience: *I have never given advice on this food item*; Discouraged: *I would discourage against this food item*.
